# PPARs in the Renal Regulation of Systemic Blood Pressure

**DOI:** 10.1155/2010/698730

**Published:** 2010-06-08

**Authors:** Tamás Rőszer, Mercedes Ricote

**Affiliations:** Department of Regenerative Cardiology, Spanish National Cardiovascular Research Center (CNIC), 28029 Madrid, Spain

## Abstract

Recent research has revealed roles for the peroxisome proliferator activated receptor (PPAR) family of transcription factors in blood pressure regulation, expanding the possible therapeutic use of PPAR ligands. PPAR*α* and PPAR*γ* modulate the renin-angiotensin-aldosterone system (RAAS), a major regulator of systemic blood pressure and interstitial fluid volume by transcriptional control of renin, angiotensinogen, angiotensin converting enzyme (ACE) and angiotensin II receptor 1 (AT-R1). Blockade of RAAS is an important therapeutic target in hypertension management and attenuates microvascular damage, glomerular inflammation and left ventricular hypertrophy in hypertensive patients and also show antidiabetic effects. The mechanisms underlying the benefits of RAAS inhibition appear to involve PPAR*γ*-regulated pathways. This review summarizes current knowledge on the role of PPARs in the transcriptional control of the RAAS and the possible use of PPAR ligands in the treatment of RAAS dependent hypertension.

## 1. Introduction

Peroxisome proliferator activated receptors (PPARs), members of the superfamily of ligand regulated transcription factors, are expressed in the cardiovascular system and control diverse vascular functions by mediating appropriate changes to gene expression (reviewed by [1, 2]). PPAR*α*, PPAR*β* */* *δ* and PPAR*γ* isoforms are expressed in endothelial cells, while vascular smooth muscle cells (VSMCs) express only PPAR*α* and PPAR*γ* [1, 3]. PPAR*α*  and PPAR*β* */* *δ* affect the vasculature through several mechanisms, including regulation of endothelial function, VSMC apoptosis, and antiinflammatory properties via the control of cytokine signaling and the downregulation of inflammatory cytokine induced genes [1, 2, 4]. Moreover, PPAR gene mutations lead to disturbances in blood pressure regulation. In humans, both the Pro12Ala polymorphism and mutations in the PPAR*γ* gene contribute to hypertension [5, 6]. Mice lacking PPAR*γ* in VSMCs or endothelial cells show altered arterial vasoconstriction [7], while PPAR*α* knockout mice develop salt-sensitive hypertension [8]. Fibrates, agonists of PPAR*α*, and thiazolidinediones (TZDs), ligands for PPAR*γ* have cholesterol- and triglyceride-lowering effects and are insulin sensitizers, with additional antiinflammatory and antiatherogenic benefits [9, 10]. These drugs are primarily used in the treatment of lipid homeostasis disorders, type 2 diabetes mellitus and atherosclerosis [1, 2]. Hypertension is a common comorbidity of atherosclerosis and insulin resistance and clinical observations show that TZDs effectively reduce blood pressure in type 2 diabetic patients [10–19]. TZDs also reduce peripheral resistance in several experimental models of hypertension leading to reduced blood pressure [20]. The antihypertensive effects of TZDs are mediated by the vascular endothelia [20, 21] and through an inhibitory action on VSMC L-type Ca^2+^-channels [9]. Endothelial function in hypertension is also improved by fibrates, and these compounds can reduce blood pressure in patients with hypertriglyceridemia [22]. Recent studies indicate that PPAR*γ*-regulated gene expression can also influence the function of the renin-angiotensin-aldosterone system (RAAS), thus allowing PPAR*γ* ligands to alter vascular tone and total body fluid volume [13–15, 23]. An involvement of PPAR*α* in the modulation of the RAAS has also been proposed, although its relevance to systemic blood pressure regulation is still disputed [10, 19, 24]. 

**Figure 1:**
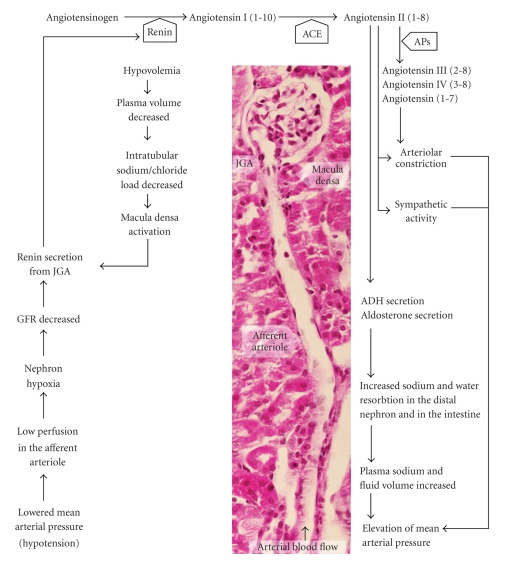
Schematic representation of the RAAS cascade. The main signals activating the RAAS are the reduced nephron perfusion due to lowered arterial mean pressure and the hypovolemia-induced decrease in intratubular sodium/chloride load. Kidney JGA cells produce renin, which activates the RAAS cascade, yielding two main effector molecules, angiotensin II (AngII) and aldosterone. Other vasoactive angiotensins can also be produced from Ang-II by the action of different aminopeptidases (APs). The main ouputs of RAAS activation are arteriolar vasoconstriction and fluid retention, which both elevate mean arterial blood pressure. PPAR*γ* is expressed in JGA, the source of renin and in the distal collecting ducts, targets of aldosterone. Histological image shows a renal glomerule with the longitudnal section of the afferent arteriole in mouse kidney (hematoxylin-eosin staining, N: 400x).

The RAAS is a cascade formed by hormone-like substances released from the kidney and the adrenal gland, and acts as a long-term regulator of systemic blood pressure and interstitial fluid volume (reviewed by [25]). The most potent effector molecule of the RAAS is angiotensin II (Ang II), which elevates systemic blood pressure through the constriction of blood vessels and furthermore enhances aldosterone secretion, catecholamine release, sympathetic nerve activity and myocardial contractility (Figure 1). Aldosterone, the other effector molecule of the RAAS, contributes to the maintenance of fluid homeostasis and blood volume through the regulation of the sodium and water resorbtion capacity of the kidneys and the intestinal epithelium [25]. Aldosterone and Ang II can also act as paracrine factors, and influence inflammation, mitogenesis, apoptosis and cell growth, through which they contribute not only to the development of hypertension but also to its cardiovascular complications [16, 25–31], such as microvascular damage, glomerular inflammation, podocyte injury and left ventricular hypertrophy [32, 33]. A major medication strategy for RAAS-dependent hypertension is therefore to block steps in the RAAS cascade, thereby lowering systemic blood pressure and reducing associated cardiovascular complications [25]. 

Newly identified roles of PPAR*γ* and PPAR*α* in the modulation of blood pressure have already begun to expand the potential therapeutic uses of PPAR*γ* and PPAR*α* ligands [15, 21]. However, the role of PPAR*α* in the regulation of the RAAS is disputed [10], and unwanted hepatotoxic and cardiac effects of currently available PPAR*γ* ligands [34, 35] limit the use of these drugs in hypertension management. A critical review of molecular mechanisms and physiological consequences of PPAR activity in the RAAS is therefore timely. In this review we summarize current knowledge about the role of PPAR*γ* and PPAR*α* in the functioning of the RAAS and discuss the possible modulation of RAAS*­*dependent hypertension by these transcription factors at the level of gene expression.

## 2. PPAR Ligands and Mechanism of Action

The three different PPAR subtypes (PPAR*α* *[*NR1C1*]*, PPAR*β* */* *δ* *[*NRC2*]* and PPAR*γ* *[*NRC3*]*) have different tissue distribution and are involved in distinct biological processes. PPAR*α* is highly expressed in liver, heart, kidney cortex and skeletal muscle; PPAR*β* */* *δ* is abundantly expressed throughout the body; and PPAR*γ* is predominantly expressed in adipose tissue, liver, kidney, skeletal muscle, monocytes and macrophages [2]. PPARs, as ligand regulated transcription factors, can modulate gene expression through binding to hormone response elements in the promoter or enhancer sequences of target genes [1, 2]. The domain strucure of PPARs is similar to that of other nuclear receptors. A ligand-independent transactivation amino-terminal domain is followed by a DNA-binding domain (DBD) that contains zinc finger motifs. The ligand binding domain (LBD) is located at the carboxy terminus and is composed of several *α* helices that form a hydrophobic ligand-binding pocket. The DBD mediates PPAR binding to PPAR response elements (PPREs) within the promoters of target genes. For DNA binding, PPARs must dimerize with another nuclear receptor, the retinoid X receptor (RXR). Heterodimers of PPARs and RXR bind to PPREs and then activate expression of gene networks involved in the control of lipid and carbohydrate metabolism in several cell types. In addition, PPARs can also transrepress proinflammatory genes by antagonizing the activities of other transcription factors such as members of the nuclear factor-*κ*B (NF-*κ*B) and activator protein-1 (AP-1) families. Several lipid mediators and a variety of molecules that are derived from fatty acid metabolism are natural ligands of PPARs. In the last decade, much attention has focused on the pharmacological modulation of PPARs. For example, fibrates (clofibrate, fenofibrate, bezafibrate) are activators of PPAR*α*, that are used in the treatment of hyeprlipidemias, and synthetic PPAR*β* */* *δ* activators can improve insulin sensitivity and increase fatty acid catabolism. TZDs, synthetic ligands of PPAR*γ*, such as rosiglitazone and pioglitazone, have already been introduced into clinical practice for the treatment of insulin resistance [1, 2]. However, animal studies with chronic TZD treatment [34, 35] and four large trials of TZDs with cardiovascular endpoints have also revealed some unwanted effects of TZDs, such as fluid retention and heart failure [34]. Today there is therefore increased interest in the identification of selective PPAR modulators (SPPARMs) to improve metabolic and antiinflammatory benefits of PPAR activation [36]. 

## 3. Renin Production Is Influenced by PPAR*γ* Ligands

Cells of the juxtaglomerular apparatus (JGA) express PPAR*γ* [37]. The JGA produces renin, a 43 kDa protease (EC 3.4.99.19, new EC 3.4.23.15) that cleaves angiotensinogen (AGT) to yield angiotensin I (Ang I). Signals that promote renin synthesis and secretion can lead to RAAS-dependent hypertension in humans [25]. Overproduction of renin can result from enhanced renin gene transcription, as occurs in spontaneously hypertensive rats, which have mutations in the transcription factor binding sites of the renin gene [38]. 

**Figure 2:**
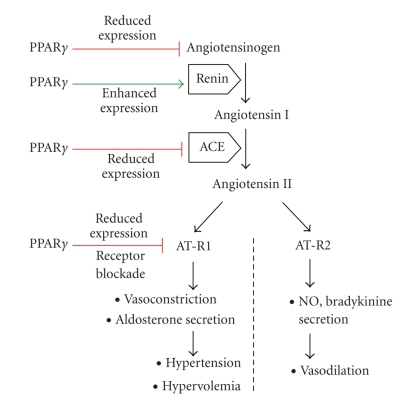
Role of PPAR*γ* in the modulation of the RAAS cascade. Gene expression of RAAS molecules is modulated by PPAR*γ* *.* Although renin production is facilitated by PPAR*γ* activation, the hypertensive effects of RAAS activation are attenuated by PPAR*γ* ligands, since these strongly reduce Ang II levels, ACE and AT-R1 gene expression. Telmisartan also exerts AT-R1 blocking properties. Simultaneously with blockade of AT-R1, the beneficial vasodilator effects of AT-R2 activation become dominant.

Endogenous and pharmacological PPAR*γ* agonists, such as unsaturated fatty acids and TZDs, have been shown to stimulate renin gene expression in renin producing cells, such as cultured JGA cells [37] (Figure 2). The hunan renin gene contains two PPAR*γ* binding sequences [37, 39] and knockdown of PPAR*γ* increases PPRE-driven renin transcription in vitro. JGA specific deletion of PPAR*γ* in mice results in upregulated renin transcription [40]. 

Although PPAR*γ* controls renin gene transcription, potential therapeutical benefits of PPAR*γ* ligand mediated changes in renin expression have yet to be evaluated. 

## 4. PPAR*α* and PPAR*γ* Regulate Angiotensinogen Gene Expression

Angiotensionogen (AGT), the first substrate of the RAAS, is an *α*_2_-glycoprotein and present at nanomolar concentrations in serum [25, 41]. Cleavage by renin yields the decapeptide Ang I, which is then converted by angiotensin converting enzyme (ACE; EC 3.4.15.1) to the active octapeptide Ang II (Figure 1). Ang II can induce arteriolar constriction and consequently elevate systemic vascular tone and blood pressure [25]. 

Elevated serum levels of AGT are often recognized as the cause of hypertension, because higher AGT concentrations lead to supernormal Ang II production [25]. Under physiological conditions, AGT is mainly synthesized in the liver, although adipocytes can also liberate physiologically relevant amounts of AGT [25–27]. In humans, hepatic AGT mRNA levels correlate positively with plasma AGT concentration [41, 42]. The human T235 allelic variant of the AGT gene is associated with enhanced hepatic AGT mRNA synthesis, higher serum AGT levels, and consequent hypertension [42]. Injection of antisense oligonucleotides against AGT into rats results in a transient fall in blood pressure accompanied by decreased levels of plasma AGT and liver AGT mRNA [43]. These data show that AGT production is tightly regulated at the level of gene expression. 

The human AGT promoter is activated by PPAR*α*/RXR heterodimers and is also bound by hepatocyte nuclear factor 4 (HNF-4), another member of the nuclear receptor family [24]. The PPAR*α* response region in the AGT promoter includes a binding site for HNF-4, composed of two core motifs (RG(G/T)TCA or a closely related sequence) separated by a single nucleotide (DR1 element). The PPAR*α* ligand bezafibrate can activate the human AGT promoter in HeLa cells, which do not express HNF-4, but this effect is not seen in HNF-4-expressing cell lines, such as HepG2 cells or HNF-4-transfected HeLa cells, indicating that HNF-4 interferes with promoter binding by PPAR*α*/RXR heterodimers [24]. These data suggest that AGT promoter activity can be enhanced by nuclear receptor signaling via PPAR*α* and RXR, predisposing to hypertension. 

**Figure 3:**
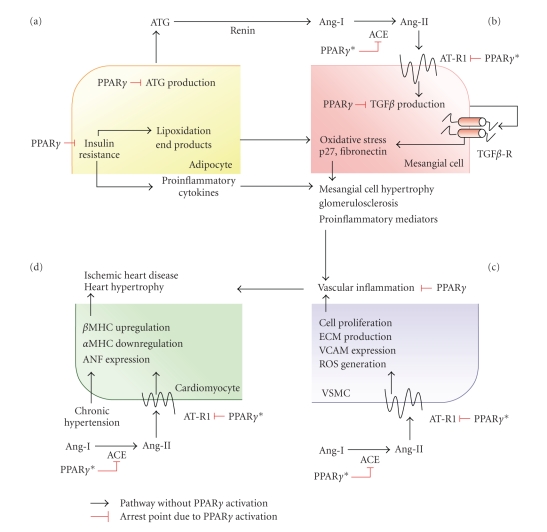
Paracrine effects of the RAAS and their modulation by PPAR*γ*. In adipocytes (a) PPAR*γ* activation is suggested to reduce ATG synthesis, impeding RAAS activation; this can ameliorate hypertension in obese diabetic patients. Independently of RAAS modulation, PPAR*γ* improves insulin action in adipocytes, reducing the production of proinflammatory cytokines and reducing the risk of atherogenesis and nephropathy. In kidney mesangial cells (b) PPAR*γ* activation reduces TGF*β*-mediated hypertrophy and downregulates expression of AT-R1 and ACE. Some PPAR*γ* ligands, such as telmisartan, combine AT-R1 blocking and ACE inhibitor properties, effectively inhibiting the proinflammatory actions of Ang-II in the kidney. This dual action can also reduce vascular inflammation resulting from VSMC dysfunction (c) and cardiomyocyte hyprtreophy in chronic hypertension (d). We labeled with asterisks PPAR*γ*, where its ligands can act through both direct AT-R1 inhibition and downregulation of ACE/AT-R1 gene expression. TGF*β*: transforming growth factor-*β*, ECM: extracellular matrix, VCAM: vascular cell adhesion molecule, ROS: reactive oxygen species, *α*MHC/*β*MHC: myosin heavy chain *α* */* *β*, ANF: atrial natriuretic factor.

In severe obesity, adipose tissue can be an important source of AGT, potentially contributing to the development of hypertension and the vascular complications of metabolic syndrome [26–29] (Figure 3). This idea is supported by work with a dominant negative P467L mutation in the ligand-binding domain of PPAR*γ*.  In humans the P467L mutation is associated with severe insulin resistance and hypertension [44] and homozygous mice with the equivalent P465L mutation die in utero [44]. Heterozygous mice are hypertensive and show increased gene expression of AGT in subcutaneous adipose tissue. Increased AGT production by white adipose tissue in this mouse model not only induces hypertension; it also increases lipogenic gene expression, leading to abnormal fat distribution [44]. The PPAR*γ* ligand rosiglitazone, however, has no effect on AGT expression in human adipocytes, and an action of PPAR*γ* on adipocyte AGT gene regulation in humans has not been demonstrated [45]. 

## 5. PPAR*γ* and PPAR*α* Modulate Expression of Angiotensin Converting Enzyme

Inhibition of ACE results in decreased production of Ang II and decreased metabolism of bradykinin, leading to systemic dilation of blood vessels and a decrease in arterial blood pressure [46]. Moreover, Ang II-induced aldosterone secretion is also reduced, leading to decreased water and sodium reabsorption and a reduction in extracellular fluid volume [25, 46]. 

Ligands of PPAR*α* and PPAR*γ* can suppress the gene expression of ACE in vascular tissues [13, 47, 48] (Figure 2). In streptozotocin-induced diabetes in rats, the PPAR*α* agonist bezafibrate and the PPAR*γ* ligand pioglitazone can equally protect against the streptozotocin-induced upregulation of ACE in the aortic wall. This action of PPARs promotes beneficial antiatherogenic effects under insulin resistant conditions [13]. Similarly, ACE gene expression in obese Zucker rats is reduced by chronic treatment with rosiglitazone [47]; and clinical studies have demonstrated that the partial PPAR*γ* agonist telmisartan inhibits ACE and blocks Ang II receptor type 1 (AT-R1) [36, 49]. This combined ACE suppressing and Ang II receptor blocking (ARB) effect strengthens the vascular protection conferred to hypertensive type 2 diabetic patients by the anti*­*inflammatory and anti*­*atherogenic consequences of PPAR*γ* activation [36, 46] (Figure 2).

In contrast, antihypertensive effect of fibrates is controversial, since although these compounds reduce blood pressure in patients with hypertriglyceridemia [22], they have the opposite effect in glucocorticoid-induced diabetes [50]. The existence of antihypertensive and antiatherogenic actions of PPAR*α* are also challenged by work with the Tsukuba hypertensive mouse (THM), a model of Ang II-induced hypertension [19]. In the THM system, transgenic expression of the entire human RAAS leads to high Ang II and aldosterone levels, causing hypertension and atherosclerosis. In PPAR*α*-deficient THM animals this hypertension is totally abolished [19], and this is accompanied by a reduction in plasma renin and by a normalization of serum aldosterone. PPAR*α*-deficient THM animals also fail to develop aortic sclerosis in response to an atherogenic diet, and the spontaneous formation of foam cells from peritoneal macrophages is also markedly reduced in these animals. This suggests that the lack of PPAR*α* protects against the oxidative stress normally seen in THM mice, possibly by reducing Ang II levels [19]. Thus these data, while confirming that PPAR*α* regulates the RAAS, indicate that PPAR*α* activation in this model aggravates hypertension and fails to protect against atherogenesis. 

## 6. Angiotensin-II Receptor Blockade and Actions of PPAR/RXR Heterodimers

Angiotensin receptor blockers (ARBs) are used to treat hypertension [25, 46]. The protective effects of ARBs are based on blockade of AT-R1s. In addition to blocking signaling downstream of AT-R1s, this blockade diverts Ang II to Ang II type 2 receptors (AT-R2s), resulting in release of the vasodilator nitric oxide (NO) [46] (Figure 2). In clinical practice, blockade of AT-R1s is often combined with ACE-inhibition, and this treatment strategy (known as double RAAS blockade) can effectively reduce blood pressure in high-risk patients [46]. The advantage of ACE inhibitors is their ability to reduce circulating and tissue Ang II levels, while ARBs potentiate the beneficial blood pressure lowering effects of bradykinin, including AT-R2 mediated generation of NO (Figure 2). 

Two ARBs, telmisartan and irbesartan, act as selective PPAR modulators (SPPARMs) [36, 49, 51, 52]. Selective PPAR*γ* modulation is a new and promising pharmacological approach, based on selective receptor-cofactor interactions and target gene regulation without unwanted PPAR*γ* side effects, such as the well-known water and sodium retention associated with TZD treatment [36, 53]. Due to its partial PPAR*γ* agonist effect, telmisartan inhibits vascular ACE activity [13], AT-R1 expression [54, 55] and increases endothelial NO synthesis [56], preventing oxidative stress and endothelial dysfunction more effectively than non PPAR*γ*-agonist ARBs [13]. Longterm treatment (4–24 weeks) with telmisartan, in monotherapy or in combination with other antihypertensive drugs reduces systolic blood pressure by 4–4.6 mmHg and diastolic blood pressure by 3–3.6 mmHg [57]. This compares favorably with the reduction of systolic blood pressure by 3 mmHg by pioglitazone in the Prospective Pioglitazone Clinical Trial in Macrovascular Events (PROactive) study [21, 58]. This degree of improvement provides sustained blood pressure control of mild to moderate hypertension [59]. In particular, the PROactive study indicates a clear clinical benefit of pioglitazone in the risk reduction of cardiovascular events in patients with type 2 diabetes, since addition of pioglitazone to conventional antihypertensive therapy reduced macrovascular outcomes by 10%, nonfatal myocardial infarction and stroke by 16% compared with placebo [60]. Pioglitazone might combine antidiabetic, antiinflammatory and antiatherogenic benefits with antihypertensive action of PPAR*γ* activation [60]. However, the recent Rosiglitazone Evaluated for Cardiac Outcomes and Regulation of glycaemia in Diabetes (RECORD) trial confirmed the increased risk of heart failure events in people treated with rosiglitazone, which limits the future use of TZDs in hypertensive patients [61]. Telmisartan was expected to provide a new therapeutic option for improved cardiovascular risk management in metabolic diseases since additionally to its ARB activity, it binds to PPAR*γ* [55]. However, neither the Ongoing Telmisartan Alone and Combination with Ramipril Global End Point Trial (ONTARGET) nor the Telmisartan Randomized Assesment Study in ACE-1 Intolerant Subjects (TRANSCEND) showed any significant advantage of telmisartan in reducing cardiovascular endpoints in high-risk patients [62]. 

PPAR*γ* also blocks the action of Ang II by transcriptionally repressing AT-R1 gene expression in VSMCs [16, 36, 49, 51, 54] (Figure 2). In addition to its role as a regulator of vascular tone, AT-R1 activation contributes to vascular lesions and atherogenesis by promoting VSMC proliferation [32, 63]; therefore a suppressed Ang II response can potentially slow the progression of atherosclerosis. VSMCs express the retinoid receptors RAR*α* and RXR*α*. In the arterial wall, long-term exposure to all-trans retinoic acid or RAR*α*/RXR*α* agonists dose-dependently inhibits Ang II-induced VSMC proliferation and the expression of c-fos and transforming growth factor-*β* (TGF*β*) mRNA, providing antiproliferative/antiinflammatory benefits and vascular protection in hypertensive patients [16, 63]. The likely mechanism of the reduced Ang II response upon retinoid receptor activation is RAR/RXR mediated downregulation of AT-R1 expression in VSMCs, similar to the action of PPAR*γ* ligands [16]. Ang-II-mediated vascular damage and endothelial dysfunction in rats are also reduced by the activation of PPAR*α*, but this effect is due to the elevated endothelial NO synthesis and reduced oxidative stress and not to AT-R1 blockade [64].

## 7. PPAR*γ* Ligands Reduce Aldosterone Levels: Vasoprotection and Cardioprotection

The main effect of aldosterone is to facilitate epithelial sodium and water resorbtion, leading to the expansion of total body fluid volume and secondarily contributing to the elevation of systemic blood pressure [25]. Permanently high aldosterone production causes microvascular injury and cardiac hypertrophy, major contributors to hypertension-associated cardiovascular morbidity and mortality [46]. 

Ang II is the main trigger of aldosterone secretion in the adrenal cortex, and AT-R1 blockers therefore reduce plasma aldosterone levels [65]. Recent studies indicate that the PPAR*γ* agonist telmisartan, which is an ARB and reduces vascular ACE activity through PPAR*γ*, is more effective at reducing aldosterone levels than non-PPAR*γ* ligand ARBs [36, 51, 65]. PPAR*γ* ligands such as rosiglitazone and PD168 cause a significant drop in blood pressure in hypertensive rats, and increase urinary aldosterone excretion [18]. The reduced heart-to-body weight ratio of ligand-treated animals indicates that PPAR*γ* activation can also diminish aldosterone-induced heart hypertrophy [18]. These results support the idea that AT-R1 blockade in combination with PPAR*γ* stimulation has a more potent aldestrerone-lowering effect than ACE-inhibition alone, and is a promising strategy for preventing the increased body fluid volume and cardiac and vascular damage induced by enhanced RAAS activity (Figure 3). 

## 8. Volume Overload is the Adverse Effect of PPAR*γ* Activation

Although PPAR*γ* stimulation reduces aldosterone levels, thereby countering water and sodium reabsorption, PPAR*γ* ligands such as rosiglitazone and PD168, concomitantly with their antihypertensive effects, cause a significant elevation of total body fluid volume in hypertensive rats [18]. Similarly, edema and water retention are frequently occurring side effects in many patients treated with TZDs [3, 34, 35, 66, 67]. This effect can normally be treated with diuretics; however, this cannot fully restore the interstitial fluid volume in TZD-treated diabetic or atherosclerotic patients if glomerulosclerosis or diabetic glomerulonephritis with osmotic diuresis has developed. Adverse metabolic effects of loop diuretics also contraindicate their administration in many cases [35, 67].

Fluid retention caused by TZDs might be a consequence of the activation of PPAR*γ* expressed in the kidney collecting ducts, since kidney epithelial PPAR*γ* positively regulates sodium and water resorbtion [68]. Other factors besides the enhanced sodium resorbtion might also be involved in the edematous side effects of PPAR*γ* ligands, since individuals with single nucleotide polymorphisms in the *β*1 adrenergic receptor gene develop peripheral edema more frequently during PPAR*γ* agonist treatment [69]. In contrast, polymorphisms in the PPAR*γ* regulated renin and endothelin-1 genes are associated with a reduced risk of water retention and edema [69]. 

Fluid retention upon PPAR*γ* ligand administration does not seem to interfere with the beneficial blood pressure lowering effects of these drugs [18]; however, four large trials of TZDs with cardiovascular endpoints have underlined the harmful side effects of PPAR*γ* activation in diabetic patients with hypertension or accompanying heart problems [34]. Although PPAR*γ* agonists reduce cardiovascular risk factors [3, 21], congestive heart failure or other ischemic heart diseases can develop unexpectedly in patients treated with TZDs [34]. Edema and fluid retention provide a possible explanation for this, since expansion of extracellular fluid volume diminishes cardiac output, triggering a compensatory cardiac hypertrophy, eventually resulting in congestive heart failure [34]. To avoid unwanted cardiac side effects, administration of TZDs is being restricted, and much attention is being directed to the development of SPPARMs in order to improve the safety of PPAR*γ* modulation [57].

## 9. RAAS Dependent Renal Injury Is a Risk Factor for Hypertension: Renoprotective PPAR*γ*

Dysfunction of the RAAS can develop into kidney disease, which can both contribute to and be exacerbated by high blood pressure and cardiovascular morbidity [70, 71] (Figure 3). The importance of RAAS blockade in the treatment of nephropathy has been clearly established, although the renal benefits of ARBs and ACE inhibitors seem to be independent of their blood pressure lowering effects [71]. Several lines of evidence indicate that the local actions of the RAAS are not only involved in the regulation of renal hemodynamics, but also play a central role in kidney inflammation and the progression of microvascular lesions [70–76]. Recent reports indicate that Ang II has proinflammatory, mitogenic and proapoptotic effects; and therefore intrarenal Ang II production is an important factor in the initiation of glomerular and tubulointerstitial inflammation, contributing to the development of nephropathy, vascular injury and hypertension [73–78]. 

Spontaneously hypertensive rats or rats with streptozotocin-induced diabetes develop significant glomerular damage, characterized by sclerosis, hypercellularity, podocyte injury with abnormal urinary protein excretion, and tubulointerstitial inflammation characterized by fibrosis, type IV collagen staining and expression of TGF*β* [47, 71, 74]. Other hallmarks of kidney disease are enhanced activity of Ang II, with increased AT-R1 expression and down-regulated expression of PPAR*γ* [71]. All these indicators of glomerular and tubulointerstitial damage can be improved by administration of ARBs [48, 71]. 

Reduction of blood pressure without RAAS blockade is, however, less effective in the mitigation of renal disease. Thus underlines the fact that kidney injury is primarily due to the inflammatory, proliferative and thrombotic effects of Ang II, which adversely affect renal perfusion and increase oxidative stress [33, 71, 75–79]. Clinical evidence suggests that telmisartan, the PPAR*γ* ligand ARB, is more potent than other ARBs or ACE inhibitors at slowing the development of nephropathy [49, 51, 76]. Telmisartan also attenuates renal damage in salt-induced hypertensive rats, possibly due to the improved endothelial NO synthase coupling and renal autoregulation [80]. Other PPAR*γ* ligands, such as TZDs, also decrease inflammatory hallmarks in the kidney, such as glomerular cell proliferation, apoptosis, and podocyte injury; moreover, these ligands can also reduce the inflammatory response to Ang II in kidney mesangial cells [13, 23, 48, 75]. The Diabetes REduction Assesment with ramipril and rosiglitazone Medication (DREAM) trial reported that RAAS blockade with the ACE inhibitor ramipril can not alter renal outcome in patients with impaired glucose tolerance and/or impaired fasting glucose levels [81], while the same study confirmed that the TZD-type PPAR*γ* ligand rosiglitazone reduces diabetic kidney complications. The mechanism of renoprotection by PPAR*γ* agonists is multifactorial, and besides antiinflammatory actions includes antifibrotic effects and suppression of the RAAS [23]. Rosiglitazone appears to reduce AT-R1 expression in the kidney, in addition to its repression of Ang II synthesis, while the ARB action of telmisartan makes this compound a promising agent that combines the classical antiinflammatory PPAR*γ* functions with a potent blood pressure lowering effect [47, 70–78, 82]. Due to its lipophilic character and long half-life, telmisartan is considered to have therapeutic potential not only in RAAS-dependent hypertension, but also in the treatment of diabetic kidney injury [70, 77, 79]. In contrast with PPAR*γ*, the possible role of PPAR*α* in the modulation of local RAAS activation in the kidney remains undefined. A recent study documented that increased PPAR*α* expression can also play a protective role in hypertensive renal injury in rats with hypertension induced by NO withdrawal and high salt diet [83]. However, although plasma RAAS activity is reduced in rats with high-salt diet induced hypertension, local RAAS activation in the kidney leads to severe renal damage [77–80]. 

## 10. Involvement of PPAR*γ* in the Effects of RAAS Blockade on Lipid and Carbohydrate Metabolism

Inhibition of the RAAS delays or prevents the development of diabetes [52, 84]. The mechanisms underlying this protective effect appear to be complex and might involve the adipose tissue RAAS, which plays an important role in the metabolism of this tissue, including regulation of the production of pro-inflammatory and antiinflammatory adipocytokines [82, 85, 86] (Figure 3). 

The regulation of PPAR*γ* expression by aldosterone and Ang-II might influence insulin sensitivity [57, 85]. In rats, chronic treatment with AT-R1 antagonist leads to hypotrophy of epididymal and retroperitoneal adipose tissue, and this is followed by reduced serum levels of leptin and increased levels of adiponectin [87]. Furthermore, AT-R1 blockade increases epididymal expression of AT-R2, fatty acid synthase and PPAR*γ*, and decreases the expression of tumor necrosis factor alpha (TNF*α*). Adipokine synthesis and reduced TNF*α* production within adipose tissue are therefore beneficial consequences of local AT-R1 inhibition, AT-R2 stimulation, and perhaps PPAR*γ* activation [87]. A recent study assessed the binding affinity of the ARBs telmisartan, valsartan and lisinopril to PPAR*γ*, finding that telmisartan is the most potent PPAR*γ* agonist [88]. In the same study, all tested ARBs increased the phosphorylation of insulin-like growth factor receptor-1 and AKT in skeletal muscle cells, and also increased the secretion of the adipokine visfatin by adipocytes [88]. In hypertensive diabetic rats telmisartan improves the metabolic profile and reduces blood pressure to normotensive values faster than the ARB valsartan or the TZD pioglitazone [89]. In addition, telmisartan increases macrophage cholesterol efflux by enhancing expression of the ATP binding cassette transporters A1 and G1 (ABCA1/G1) and of scavenger receptor class B type I, and these effects are dependent on PPAR*γ* regulated pathways [90]. The obesity-associated decrease in adiponectin and the increase in proinflammatory adipokines are linked to the development of insulin resistance, which is coupled to altered macrophage lipid metabolism, enhanced atherogenesis; and hypertension [1, 2]. The consequences of PPAR*γ* activation on insulin signaling, adiponectin and adipokine gene expression, as well as macrophage cholesterol handling, might underlie the mechanism of the additional antidiabetic and vasoporotective effects of AT-R1 blockade.

The metabolic benefits of RAAS blockade have been challenged by the DREAM trial. This study shows that the ACE inhibitor ramipril does not reduce new-onset diabetes, although it does significantly increase the regression to normogylcemia, suggesting beneficial effects on glucose homeostasis [91]. The DREAM trial involved a relatively young patient population with moderate hypertension and without cardiovascular disease, which might explain the lack of an effect on new-onset diabetes [81, 91]. The question of whether the prevention of new-onset diabetes leads to a reduction in cardiovascular disease events might be resolved by the results of two ongoing clinical trials, Nateglinide And Valsartan in Impaired Glucose Tolerance Outcomes Research (NAVIGATOR) and ACE Inhibitor-based versus Diuretic-based Antihypertensive Primary Treatment in Patients with PreDiabetes (ADaPT) [62]. 

## 11. Conclusion and Outlook

Gene expression of RAAS molecules is modulated by PPAR*γ*, and to a lesser extent by PPAR*α*, making the ligands of these transcription factors potential blood pressure modulating drugs in RAAS-dependent hypertension [3, 21, 70]. The blood pressure reduction achieved by PPAR*γ* ligands suggests that PPAR*γ* activation can be used in the long-term control of moderate hypertension [92]. Nephropathy, a predisposing risk factor for hypertension, can be also ameliorated by PPAR*γ* and PPAR*α*, athough these renoprotective effects are due to antiinflammatory and vasoprotective PPAR*γ*/PPAR*α* properties and the reduction in the proinflammatory Ang II effects, and are independent of the systemic RAAS modulation. Transcription of angiotensinogen seems also to be regulated by PPAR*α*, although the value of PPAR*α* as a therapeutic target in RAAS dependent hypertension should be evaluated. 

Since activation of PPAR*γ* modulates both the systemic and paracrine effects of the RAAS, selective PPAR*γ* modulators may provide blood pressure lowering benefits with synergistic vasoprotective, renoprotective and cardioprotective consequences. However, TZD-type PPAR*γ* ligands disturb water and salt homeostasis in susceptible individuals, leading to adverse cardiac side effects, which is today a strong limitation for the use and future impact of the currently available PPAR*γ* activators. The development of SPPARMs and further studies in mice with tissue specific deletion of PPAR isoforms will facilitate better understanding and pharmacological modulation of nuclear receptor functions in hypertension. 

## Abbreviations

ACE: Angiotensin I converting enzyme
Ang I: Angiotensin I
Ang II: Angiotensin II
AT-R1: Angiotensin II receptor type 1
ADH: Antidiuretic hormone
ANF: Atrial natriuretic factor
AT-R2: Angiotensin II receptor type 2
ATG: Angiotensinogen
ARB: Angiotensin II type 1 receptor blocker
JGA: Juxtaglomerular apparatus
RAAS: Renin-angiotensin-aldosterone system
SPPARM: Selective PPAR modulator
VSMC: Vascular smooth muscle cell.
